# A Healthy Diet

**Published:** 1886-05

**Authors:** 


**A Healthy Diet.**—The following from the *New England Farmer* contains at least some good. The custom is becoming so common in the towns and villages, at hotels as well as private houses to have for breakfast, oatmeal, wheaten grits, or other cereals, with graham "gems" and fruit, so that the traveller or guest may choose between such diet and the usual meat and hot cake food, that our farming communities must fall into the habit sooner or later, and give their children at least a chance to work out their own salvation. How much clearer is their head, how much less craving the appetite for drinks and stimulants, how more under subjection their temper, and how more healthful their whole system, when the food is mainly of an unexciting nature, and how soon the taste is formed to enjoy it, and to cease to crave after the fleshpots which have heretofore yielded their more noxious supplies. There are many farmers and their wives who are considering these things, but hesitate about differing from their neighbors, or are, as is too common in this country, afraid of their children; but let them once more try a change and have their morning and evening meals consist of grains and fruit, with well-baked bread, and not always fresh and hot, and such vegetables as they desire, and milk for the children, water and tea for coffee, and see if, after a sufficient length of time to produce effects, there is not more health, peace and contentment in the household, and a consciousness that the way is not being prepared for subsequent violences and breaches of nature's and man's laws occasioned by gross appetites and indulgences.

**Choking.**—A baby or young child may hold its breath while there is food in the mouth, simply because it cannot obtain more food or cannot have its own way. As soon as the spasm of the muscles of the throat relaxes, an inspiration occurs, air is forcibly drawn into the lungs, and if particles of food have not already been removed from the mouth and throat by one's finger they are likely to block up the larynx and cause suffocation. In other words, they are "foreign bodies." Children just passing out of babyhood who are allowed to feed themselves at table and to eat whatever they want run great risks of suffocation by large mouthfuls of food. No careful parent who has repeatedly observed a baby's manner of cramming the mouth full and of gulping food, if left to himself, doubts that suffocation may thereby be caused. To reduce the danger to the minimum, therefore, additional food should not be given until the baby's mouth is quite empty, and the mother should not entrust the feeding to other hands than her own, unless, indeed, she intelligently supervises it.—*Dr. Jerome Walker, in Babyhood.*

